# miR-3189-3p Mimics Enhance the Effects of *S100A4* siRNA on the Inhibition of Proliferation and Migration of Gastric Cancer Cells by Targeting *CFL2*

**DOI:** 10.3390/ijms19010236

**Published:** 2018-01-13

**Authors:** Yue Bian, Junfu Guo, Linlin Qiao, Xiuju Sun

**Affiliations:** 1 Department of Medical Genetics, China Medical University, Shenyang 110122, China; bianyue@cmu.edu.cn (Y.B.); guojunfu@aliyun.com (J.G.); llqiao@cmu.edu.cn (L.Q.); 2 Teaching and Experiment Center, Liaoning University of Traditional Chinese Medicine, Shenyang110847, China

**Keywords:** gastric cancer, *S100A4*, miR-3189-3p, *CFL2*, proliferation, migration

## Abstract

*GDF15* is a downstream gene of *S100A4*. miR-3189 is embedded in the intron of *GDF15*—and coexpressed with it. miR-3189-3p functions to inhibit the proliferation and migration of glioblastoma cells. We speculated that *S100A4* might regulate miR-3189-3p to affect its function in gastric cancer cells. Quantitative reverse transcription polymerase chain reaction (qRT-PCR) showed that miR-3189-3p expression was significantly downregulated in MGC803 cells after *S100A4* knockdown. Overexpression of miR-3189-3p significantly inhibited the proliferation and migration of the cells. Moreover, miR-3189-3p mimics enhanced the effects of an *S100A4* siRNA on the inhibition of cell proliferation and migration. Dual luciferase reporter assays, qRT-PCR, and Western blotting verified that *CFL2* is a direct target of miR-3189-3p. *CFL2* mediates the regulation of miR-3189-3p on the proliferation and migration of MGC803 cells. Data mining based on Kaplan–Meier plots showed that high *CFL2* expression is associated with poor overall survival and first progression in gastric cancer. These data suggested that miR-3189-3p mimics enhanced the effects of the *S100A4* siRNA on the inhibition of gastric cancer cell proliferation and migration by targeting *CFL2*. The findings suggested that when targeting *S100A4* to treat gastric cancer, consideration and correction for counteracting factors should obtain a satisfactory effect.

## 1. Introduction

Gastric cancer (GC) is the fifth most common malignancy worldwide [1]. Although the incidence of GC has declined, the overall 5-year survival rate for patients with GC remains unsatisfactory, largely because GC is almost always diagnosed at advanced stages, and the effectiveness of current treatment is limited. Therefore, determining the molecular mechanisms that affect the properties of GC cells would be helpful to identify biomarkers for the early detection of gastric cancer and to develop novel therapeutic targets to treat GC at the molecular level, therefore improving the prognosis of patients with GC.

*S100A4* encodes a member of the S100 family of calcium-binding proteins. Accumulating evidence shows that it plays very important roles in the progression and metastatic potential of various types of cancers, including GC [2], lung cancer, colorectal cancer, cervical cancer, and breast cancer [3,4,5,6]. Our previous studies showed that *S100A4* suppression by RNA interference (RNAi) could inhibit the proliferation and migration of GC cells, and promote their anoikis [7,8,9]. To investigate the underlying mechanism by which *S100A4* affects the properties of GC cells, we analyzed the differentially expressed gene profile using a cDNA microarray after *S100A4* suppression in GC cell line MGC803, and found that *GDF15* was significantly downregulated among the 173 differentially expressed genes, and could mediate the effect of *S100A4* on the properties of GC cells [10].

MicroRNAs (miRNAs) are non-coding, short (20–22 nt) RNA molecules that can cause translation repression and/or mRNA degradation by binding to the 3′-untranslated regions (3′-UTRs) of target mRNAs. Many studies have demonstrated that miRNAs play crucial roles in the development and progression of human cancers [11,12,13]. It had been suggested that miRNAs such as miR-646, miR-381, miR-154, miR-133b, and miR-93-5p are key regulators of the proliferation, invasion, and migration of GC cells [14,15,16,17,18]. They can also serve as potential biomarkers and therapeutic targets in GC. miR-3189 is a novel primate-specific miRNA embedded in the intron of the *GDF15* gene. miR-3189-3p could inhibit cell proliferation and/or migration in colorectal cancer cells [19] and glioblastoma cells [20]. In addition, miR-3189 showed potential diagnostic value in cholangiocarcinoma and oral cancer [21,22]. However, microRNA array analysis demonstrated that miR-3189-3p was one of the most highly upregulated miRNAs in microdissected prostate cancer in comparison with the matched neighboring normal prostate epithelium [23]. These findings indicated that the functional roles of miR-3189-3p in human cancers might vary between different types of cancer. Until now, the expression status and function of miR-3189-3p in GC cells remained unknown. We showed that *S100A4* inhibition leads to significantly decreased expression of *GDF15*; therefore, we speculated that *S100A4* might regulate the expression of miR-3189-3p, which lies in the intron of *GDF15*. In addition, miR-3189-3p might affect the role of *S100A4* inhibition on the properties of GC cells.

In this study, we found that miR-3189-3p was downregulated in MGC803 cells after *S100A4* knockdown. Functionally, we found that miR-3189-3p mimics could significantly inhibit the proliferation and migration of MGC803 cells. miR-3189-3p mimics enhanced the effects of *S100A4* siRNA in inhibiting the proliferation and migration of MGC803 cells. Moreover, a dual luciferase reporter assay verified that *CFL2* is a direct target of miR-3189-3p. Functional analysis indicated that *CFL2* mediates the regulation of miR-3189-3p in the proliferation and migration of MGC803 cells. In addition, Kaplan–Meier plot analysis revealed that high *CFL2* expression is closely related to unfavorable overall survival (OS) and first progression (FP) in patients with GC.

## 2. Results

### 2.1. S100A4 Knockdown Leads to Decreased Expression of miR-3189-3p in MGC803 Cells

**Figure 1:**
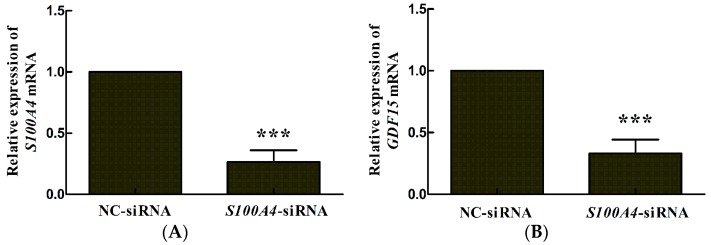

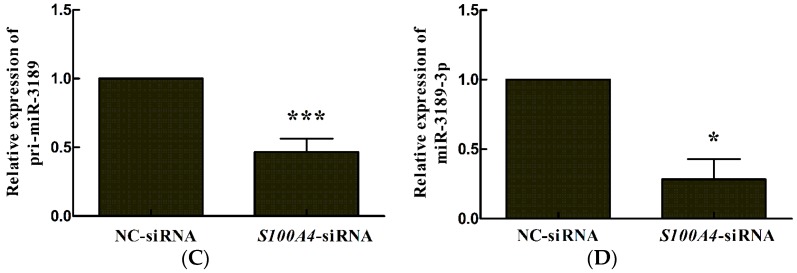
*S100A4* knockdown leads to decreased expression of miR-3189-3p in MGC803 cells. MGC803 cells were transfected with either *S100A4*-siRNA (small interfering RNA) or NC-siRNA, mRNA was extracted for quantitative reverse transcription polymerase chain reaction (qRT-PCR) analysis of (**A**) *S100A4,* (**B**) *GDF15*, (**C**) pri-miR-3189, and (**D**) miR-3189-3p at 48 h after transfection. Data represent the mean of three independent experiments. *GAPDH* was used for the internal control. * *p* < 0.05, *** *p* < 0.001. NC: negative control.

Previous studies by our group showed that *GDF15* is an important downstream gene of *S100A4*. Other researchers reported that miR-3189 is an intronic miRNA of *GDF15*. Therefore, we hypothesized that *S100A4* could also regulate miR-3189 expression. The results from quantitative reverse transcription polymerase chain reaction (qRT-PCR) showed that after *S100A4* inhibition by RNA interference (Figure 1A), *GDF15* expression was downregulated (Figure 1B), as reported by our previous study [10]. Furthermore, pri-miR-3189 and miR-3189-3p were both significantly downregulated after *S100A4* inhibition (Figure 1C,D), which indicated that *S100A4* could regulate miR-3189-3p expression in MGC803 cells.

### 2.2. miR-3189-3p Inhibits the Proliferation of MGC803 Cells

**Figure 2:**
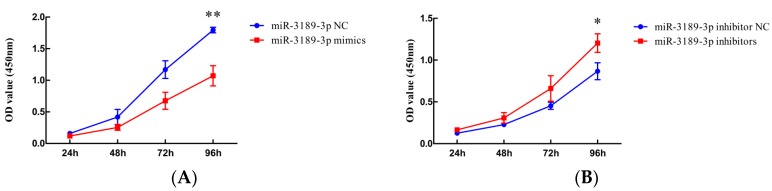
The effect of miR-3189-3p on MGC803 cell proliferation. MGC803 cells were transfected with miR-3189-3p inhibitor NC, miR-3189-3p inhibitors, miR-3189-3p NC, or miR-3189-3p mimics respectively. The Cell Counting Kit-8 (CCK8) assay was used to examine the effect of miR-3189-3p (**A**) mimics or (**B**) inhibitors on MGC803 cell proliferation. The data represent the mean ± SD from three independent experiments. * *p* < 0.05, ** *p* < 0.01.

The results from the Cell Counting Kit-8 (CCK8) assay showed that at 96 h after transfection of miR-3189-3p mimics, the proliferation of MGC803 cells was significantly decreased compared with cells transfected with miR-3189-3p negative control (NC) (*p* < 0.01) (Figure 2A). Meanwhile, miR-3189-3p inhibitors led to increased proliferation of MGC803 cells compared to cells treated with inhibitor NC at 96 h after transfection (*p* < 0.05) (Figure 2B). These findings demonstrated that miR-3189-3p could inhibit the proliferation of MGC803 cells.

### 2.3. miR-3189-3p Retards MGC803 Cells Migration

**Figure 3:**
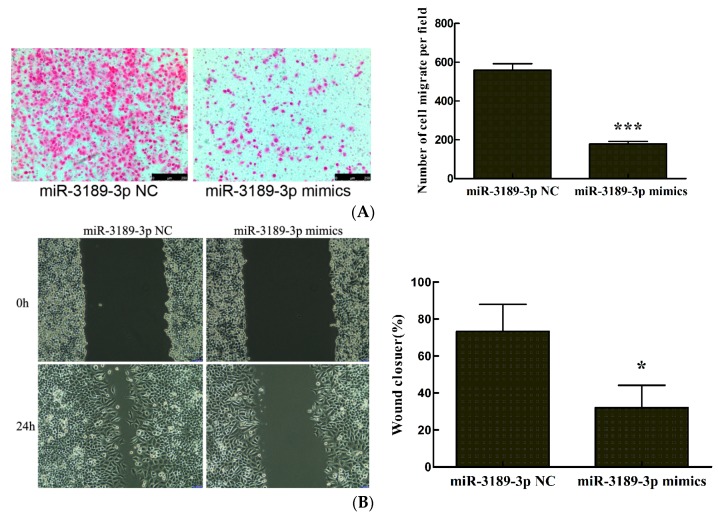
The effect of miR-3189-3p on MGC803 cell migration. The detection of MGC803 cell migration by the (**A**) transwell or (**B**) wound healing assays after miR-3189-3p mimics transfection. (left, magnifcation, ×100). All the results were obtained from three independent experiments. * *p* < 0.05, *** *p* < 0.001.

We further investigated the effects of miR-3189-3p on the migration of MGC803 cells by performing transwell and wound healing assays. The results from the transwell assay demonstrated that the number of migrated cells significantly decreased after transfection with miR-3189-3p mimics compared with the cells transfected with the negative control (*p* < 0.001) (Figure 3A). The results from the wound healing assays demonstrated that the wounds were significantly wider for the cells transfected with miR-3189-3p mimics compared with the cells transfected with the negative control (*p* < 0.05) (Figure 3B). These results indicated that miR-3189-3p could inhibit the migration of MGC803 cells.

### 2.4. miR-3189-3p Mimics Enhanced the Effects of S100A4 siRNA on the Inhibition of Proliferation and Migration of MGC803 Cells

**Figure 4:**
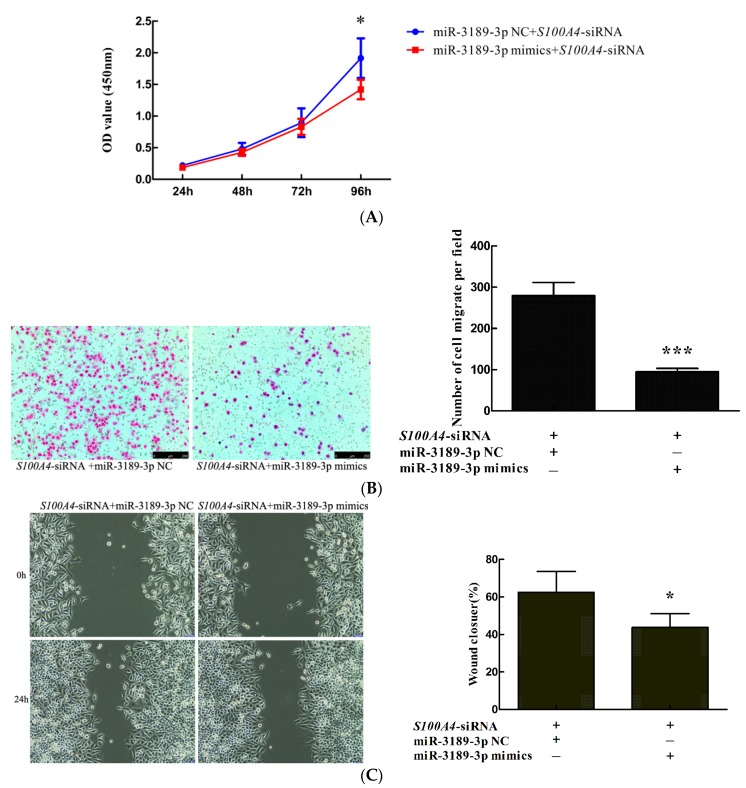
miR-3189-3p mimics enhanced the effects of *S100A4* small interfering RNA (siRNA) on the inhibition of the proliferation and migration of MGC803 cells. MGC803 cells were co-transfected with *S100A4*-siRNA and miR-3189-3p mimics or miR-3189-3p NC. The effect on proliferation or migration was analyzed by (**A**) CCK8, (**B**) transwell, and (**C**) wound healing assays. (**B**,**C**) left, magnifcation, ×100. All the results were obtained from three independent experiments. * *p* < 0.05, *** *p* < 0.001.

We observed that *S100A4* inhibition by RNAi led to the downregulation of miR-3189-3p. In addition, we demonstrated that miR-3189-3p mimics could inhibit the proliferation and migration of MGC803 cells. Our previous research showed that *S100A4* inhibition by RNAi led to decreased proliferation and migration of MGC803 cells. Therefore, we speculated that miR-3189-3p could counteract the effect of *S100A4* and that miR-3189-3p mimics would enhance the effect of *S100A4* siRNA on the inhibition of the proliferation and migration of MGC803 cells. To validate this hypothesis, we co-transfected *S100A4*-siRNA with miR-3189-3p mimics or miR-3189-3p NC into MGC803 cells and detected the proliferation and migration of the cells using CCK-8, transwell, and wound healing assays, respectively. The results showed that the proliferation of MGC803/*S100A4*-siRNA+miR-3189-3p mimics was significantly lower than that of MGC803/*S100A4*-siRNA+miR-3189-3p NC cells (Figure 4A). The results from transwell and wound healing assays showed that the migration of MGC803/*S100A4*-siRNA+miR-3189-3p mimics cells was significantly less than that of MGC803/*S100A4*-siRNA+miR-3189-3p NC cells (Figure 4B,C), suggesting that miR-3189-3p mimics enhanced the effects of *S100A4* siRNA on the inhibition of the proliferation and migration of MGC803 cells.

### 2.5. CFL2 Is a Direct Target Gene of miR-3189-3p in MGC803 Cells

**Figure 5:**
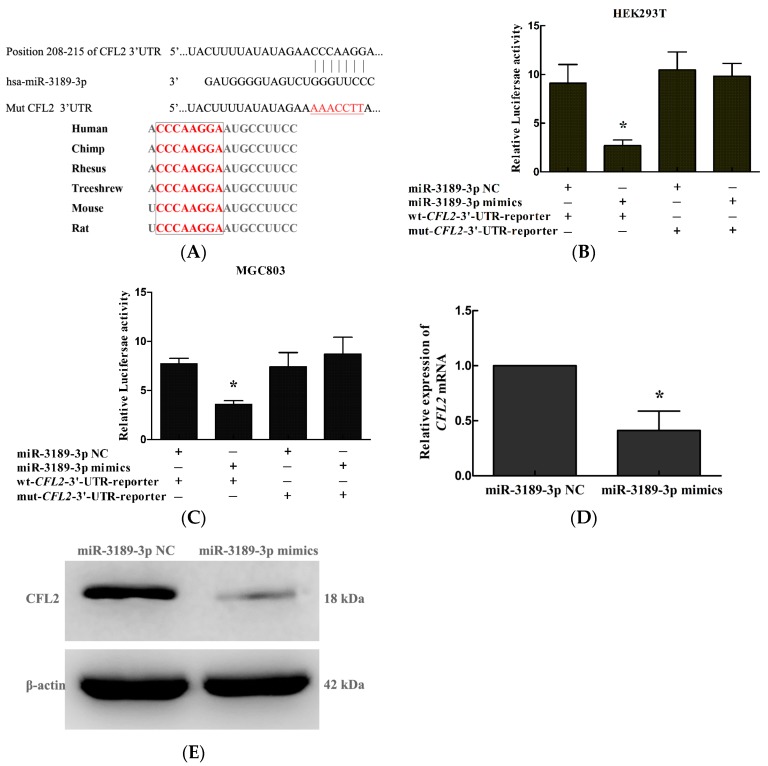
*CFL2* is a direct target gene of miR-3189-3p. (**A**) The putative miR-3189-3p binding sites in the *CFL2* 3′ untranslated region (3′-UTR) ,the sequences marked in red colour inside the box indicate seed sequences of miR-3189-3p which are conserved among different species. The underlined sequence ‘AAACCTT’ marked in red colour refers to the mutated bases in the sequence used to construct the mut-*CFL2*-3′-UTR-reporter vector. Luciferase activity in (**B**) HEK293T and (**C**) MGC803 cells after co-transfection of miR-3189-3p mimics or negative control with wt-*CFL2*-3′-UTR-reporter or mut-*CFL2*-3′-UTR-reporter constructs. (**D**) Quantitative reverse transcription polymerase chain reaction (qRT-PCR) and (**E**) Western blotting were used to detect *CFL2* mRNA and protein expression in MGC803 cells after transfection with the miR-3189-3p mimics or negative control. Data were from three independent experiments and are presented as the mean ± SD. * *p* < 0.05.

To study the underlying mechanism for the functional effect of miR-3189-3p, we searched for candidate target genes of miR-3189-3p using miRanda (http://www.microrna.org/) and TargetScan (http://www.targetscan.org). We found that the 3′ UTR of *CFL2* (cofilin 2) contained a binding site for miR-3189-3p which was conserved among many species, for example, human, chimp, and rhesus, suggesting that *CFL2* is a candidate target gene of miR-3189-3p (Figure 5A). To confirm whether miR-3189-3p directly targets the 3′ UTR of *CFL2*, we carried out a dual-luciferase reporter gene assay. As shown in Figure 5B,C, overexpression of miR-3189-3p significantly suppressed the luciferase activity of the wt-*CFL2*-3′-UTR-reporter, but not that of mut-*CFL2*-3′-UTR-reporter in HEK293T cells or MGC803 cells. Furthermore, qRT-PCR and Western blotting showed that overexpression of miR-3189-3p significantly decreased the mRNA (Figure 5D) and protein (Figure 5E) levels of *CFL2* in MGC803 cells. Taken together, the data indicated that *CFL2* is a downstream target gene of miR-3189-3p in GC cells.

### 2.6. CFL2 siRNA Inhibits the Proliferation and Migration of MGC803 Cells

**Figure 6:**
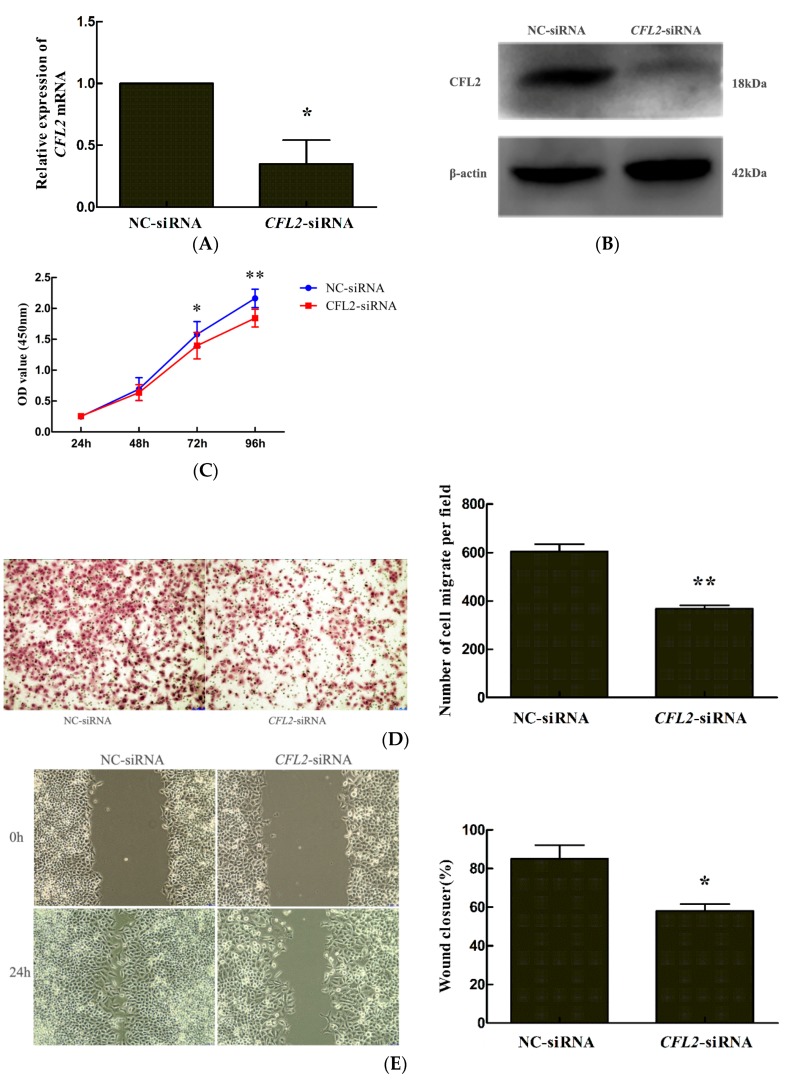
*CFL2* inhibits the proliferation and migration of MGC803 cells. (**A**) Quantitative reverse transcription polymerase chain reaction (qRT-PCR) and (**B**) Western blotting were used to detect *CFL2* mRNA and protein expression in MGC803 cells after transfecting with *CFL2*-siRNA or NC-siRNA. The effect of *CFL2* knockdown on the proliferation and migration of MGC803 cells was examined by (**C**) CCK8, (**D**) transwell, and (**E**) wound healing assays, respectively. (**D**,**E**) left, magnifcation, ×100. Data are from three independent experiments. * *p* < 0.05, ** *p* < 0.01.

We investigated the effect of *CFL2* on the biological properties of MGC803 cells. The results showed that transfection of a *CFL2*-siRNA significantly reduced *CFL2* mRNA (Figure 6A) and protein (Figure 6B) levels in MGC803 cells, indicating that the *CFL2*-siRNA could efficiently inhibit *CFL2* expression in the cells. Functionally, CCK8, transwell, and wound healing assays showed that the cell proliferation (at 72 and 96 h after transfection) and migration ability of MGC803 cells were significantly decreased after *CFL2* inhibition in MGC803 cells (Figure 6C–E). These findings suggested that *CFL2* plays an important role in regulating the proliferation and migration of MGC803 cells.

### 2.7. CFL2 Mediates the Functional Effects of miR-3189-3p on MGC803 Cells.

**Figure 7:**
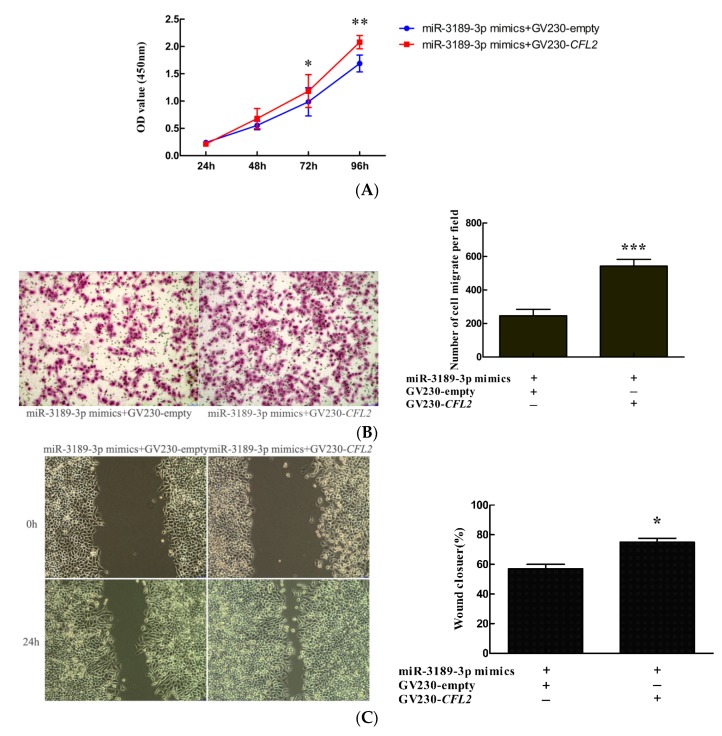
*CFL2* mediated the functional effects of miR-3189-3p on MGC803 cells. The effects of co-transfection of GV230-*CFL2* vector or GV230-empty vector with miR-3189-3p mimics on the proliferation and migration of MGC803 cells were detected by (**A**) CCK8, (**B**) transwell, and (**C**) wound healing assays, respectively. (**B**,**C**) left, magnifcation, ×100. Data are from three independent experiments. * *p* < 0.05, ** *p* < 0.01, *** *p* < 0.001.

To further explore whether *CFL2* could mediate the biological function of miR-3189-3p in GC cells, we co-transfected miR-3189-3p mimics and the *CFL2* expression vector GV230-*CFL2* into MGC803 cells, with the co-transfection of miR-3189-3p mimics and the GV230-empty vector as a control. The results showed that *CFL2* overexpression attenuated the inhibitory effects of miR-3189-3p mimics on the proliferation (Figure 7A) and migration (Figure 7B,C) of MGC803 cells. These data suggested that as a downstream target, *CFL2* could mediate the functional effect of miR-3189-3p in MGC803 GC cells.

### 2.8. CFL2 Is an Unfavorable Prognostic Factor for Gastric Cancer

**Figure 8:**
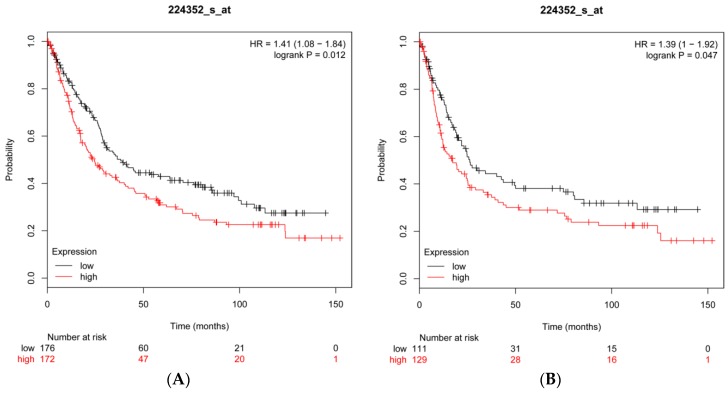
Kaplan–Meier survival curves generated from the Kaplan–Meier plotter platform for *CFL2* mRNA expression in patients with gastric cancer. (**A**) Overall survival (OS) curves for patients with gastric cancer. (**B**) First progression (FP) curves for patients with gastric cancer. HR: hazard ratio, 95% CI: 95% confidence interval.

To further investigate the prognostic value of *CFL2*, we performed data mining in the Kaplan–Meier plotter platform and found that high *CFL2* mRNA expression is associated with worse OS (hazard ratio (HR) = 1.41; 95% confidence interval (CI): 1.08–1.84; *p* = 0.012) (Figure 8A) and worse FP (HR = 1.39; 95% CI: 1–1.92; *p* = 0.047) (Figure 8B) for patients with GC, suggesting that *CFL2* is an unfavorable prognostic factor for GC.

## 3. Discussion

MicroRNAs (miRNAs) are small non-coding endogenous RNAs. According to their genomic locations, miRNAs are classified as intergenic miRNAs or intragenic miRNAs. Intergenic miRNAs lie between protein-coding genes. Intragenic miRNAs are embedded within host genes. Approximately half of the known vertebrate miRNAs are located in the introns of host genes, and are termed intronic miRNAs [24]. If they share common promoters with their respective host genes, intronic miRNAs could be co-regulated with host genes such as miR-107/*PANK1* and miR-9-1/*CROC-4* [25,26].

It has recently been reported that miR-3189 is located at the intron of the *GDF15* gene. *GDF15* and miR-3189-3p are transcriptionally co-regulated by *p53* [19]. We have found that *S100A4* inhibition by RNAi led to significantly decreased *GDF15* expression [10]; therefore, we speculated that miR-3189-3p might also be regulated by *S100A4*. In this study, we found that pri-miR-3189 and miR-3189-3p were both downregulated in MGC803 GC cells after *S100A4* inhibition, suggesting that miR-3189-3p is a downstream component of *S100A4* and is regulated by it. We then investigated the functional significance of the regulation of *S100A4* on miR-3189-3p.

Previously, Jones [19] demonstrated that miR-3189-3p could inhibit the proliferation of colorectal cancer cells. In astrocytoma and glioblastoma, the expression of miR-3189-3p was downregulated and miR-3189-3p overexpression significantly inhibited the proliferation and migration of the tumor cells [20]. However, miR-3189-3p’s function in GC cells has not been reported. Thus, we first investigated the effect of miR-3189-3p on the properties of GC cells. The results showed that overexpression of miR-3189-3p significantly decreased the proliferation and migration of MGC803 cells compared with the negative control, suggesting that miR-3189-3p could inhibit the proliferation and migration of GC cells. These findings were consistent with previous reports on colorectal cancer cells, astrocytoma, and glioblastoma cells [19,20], indicating that miR-3189-3p may also act as a tumor suppressor in gastric cancer.

RNAi was used to knock down *S100A4* to study its effects on the properties of cancer cells. Specific knockdown of *S100A4* resulted in cell responses in human GC and other cancer cells, such as decreased proliferation, migration, and invasion [7,9,27,28,29]. At the molecular level, the response involved a change in the expression of many genes. After *S100A4* knockdown in cancer cells, certain genes that inhibit proliferation and migration (e.g., *FAM107B* and *E-cadherin*) were upregulated [27,30], while those genes that normally promote proliferation and migration (e.g., *NF-κB, p65 and MMP2*) were downregulated [7,31]. In addition, ectopic overexpression of *S100A4* led to upregulation of oncogenic microRNA (miR)-155 expression in hepatocellular carcinoma cells, and an miR-155 inhibitor significantly attenuated the invasion-promoting effects of *S100A4* [32]. The above findings showed that the consequences of the resulting alterations in downstream gene expression were consistent with the overall effect of *S100A4* knockdown on the properties of cancer cells, and that these downstream genes mediated the effect of *S100A4* on the properties of cancer cells. Interestingly, in the present study, *S100A4* knockdown led to reduced expression of miR-3189-3p, which inhibited the proliferation and migration of MGC803 GC cells. We supposed that the downregulation of miR-3189-3p might attenuate/counteract the inhibitory effect of *S100A4* knockdown on the malignancy of cancer cells. To validate our hypothesis, we compared the effect of *S100A4* siRNA and miR-3189-3p mimics co-transfection with that of *S100A4* siRNA and negative control co-transfection. The results showed that the proliferation and migration of MGC803/*S100A4*-siRNA+miR-3189-3p mimic cells was significantly lower than that of MGC803/*S100A4*-siRNA+miR-3189-3p NC cells. The results indicated that the reduced miR-3189-3p expression could attenuate/counteract the inhibitory effects of S100A4 knockdown on the malignancy of cancer cells, while miR-3189-3p mimics enhanced the effects. These data provide new clues for targeting *S100A4* in cancer treatment, indicating that in order to obtain an ideal inhibitory effect of *S100A4* blockade on the malignancy of cancer cells, we should pay attention to its downstream counteracting mechanisms and try to correct them. A previous study reported a similar phenomenon in which *KRAS* inhibition led to a dramatic upregulation of ribosomal proteins L26 and L29 (*RPL26* and *RPL29*), while knockdown of *RPL26* or *RPL29* expression significantly suppressed cell proliferation of pancreatic cancer, suggesting that upregulation of *RPL2*6 or *RPL29* might counteract the effect of *KRAS* silencing in pancreatic cancer cells [33].

To better understand the molecular mechanism by which miR-3189-3p is responsible for the anti-tumor effects in GC cells, we further studied the target gene of miR-3189-3p in MGC803 cells. It has been reported that miR-3189-3p inhibits the proliferation and migration of colon cancer cells by targeting *SF3B2* and *p63RhoGEF*, respectively [19]. It is well known that miRNAs usually target many different genes to exert their functions. Therefore, we tried to search for new target genes of miR-3189-3p in MGC803 cells. By searching miRanda and TargetScan, we identified *CFL2* as a candidate target gene of miR-3189-3p. Through dual-luciferase reporter gene assays, qRT-PCR, and Western blotting, we confirmed that *CFL2* is a target gene of miR-3189-3p in MGC803 cells.

*CFL2* encodes cofilin-2 protein, which is a member of the ADF/cofilins family of actin-binding proteins. By analyzing the structure of CFL2, Yehl et al. [34] found that human CFL2 could bind to F-actin and played an essential role in accelerating actin treadmilling. Schwickert et al. [35] confirmed that *CFL2* is a target gene of miR-142-3p and is involved in regulating breast cancer invasiveness. Similarly, Luo et al. [36] demonstrated that *CFL2* was overexpressed in aggressive breast cancer cell lines and acted as a target gene of miR-200c. Knockdown of *CFL2* inhibited cell migration and invasion. Tissue microarray analysis showed that *CFL2* expression in breast cancer tissue was positively correlated with tumor grade. In contrast, in pancreatic cancer, *CFL2* showed lower expression compared with non-cancerous tissues [37]. The overexpression of *CFL2* gene in glioblastoma multiforme contributes to increased therapeutic response [38]. These data suggested that the role of *CFL2* varies in different kinds of cancers. The role of *CFL2* in GC cells has not been reported. In the present study, we showed that *CFL2* inhibition by siRNA significantly inhibited the proliferation and migration of MGC803 cells. Overexpression of *CFL2* reversed the reduced cell proliferation and migration induced by the transfection of miR-3189-3p mimics, suggesting that miR-3189-3p suppressed the proliferation and migration of gastric cancer cells by directly inhibiting *CFL2* expression. Additionally, by data mining in the Kaplan–Meier plotter, we found that high *CFL2* expression was associated with poor OS (overall survival) and FP (first progression) in patients with GC, suggesting that *CFL2* was an unfavorable prognostic factor for gastric cancer.

In conclusion, we found that *S100A4* inhibition significantly decreased the expression of miR-3189-3p in MGC803 cells. miR-3189-3p mimics inhibited the proliferation and migration of MGC803 gastric cancer cells, suggesting that miR-3189-3p acts as a tumor suppressor in the cells. miR-3189-3p mimics enhanced the effect of *S100A4* siRNA on the inhibition of cell proliferation and migration, suggesting that the reduction of miR-3189-3p attenuated the inhibitory effect of *S100A4* blockade on the properties of GC cells. *CFL2* was identified as a target of miR-3189-3p and was downregulated by miR-3189-3p. *CFL2* mediated the regulation of miR-3189-3p of the proliferation and migration of GC cells. High *CFL2* expression is associated with poor prognosis in patients with GC. These data established the connection among *S100A4*, miR-3189-3p, and *CFL2* in GC cells, demonstrating that miR-3189-3p mimics enhanced the effects of *S100A4* siRNA on the inhibition of the proliferation and migration of MGC803 GC cells by targeting *CFL2*. Overall, our findings suggested that in order to obtain an ideal effect when using *S100A4* as a target to treat gastric cancer, attention should be paid to the counteract factors.

## 4. Materials and Methods

### 4.1. Cell Culture

Human gastric cell line MGC803 was purchased from the Cell Resource Center, Institute of Basic Medical Sciences (IBMS, Beijing, China), Chinese Academy of Medical Sciences and Peking Union Medical College (CAMS/PUMC, Beijing, China). The cell line was maintained in Dulbecco’s modified Eagle’s medium (DMEM) (Invitrogen, Carlsbad, CA, USA) supplemented with 10% fetal bovine serum at 37 °C, 5% CO_2_. Human embryonic kidney cells HEK293T were obtained from the KeyGEN BioTECH Company of Jiangsu Province and were maintained in Roswell Park Memorial Institute (RPMI) 1640 medium (GIBCO, Los Angeles, CA, USA) with 10% new-born calf serum (Hyclone, Logan, UT, USA) at 37 °C, 5% CO_2_.

### 4.2. Cell Transfection

The duplex siRNA oligonucleotides specific for human *S100A4* or *CFL2*, and oligonucleotides specific for hsa-miR-3189-3p were synthesized by GenePharma (Shanghai, China), and are listed in Table 1. MGC803 cells were transfected with a final concentration of 50 nM of the specific siRNA, or 40 nM of miR-3189-3p oligonucleotides, respectively, using Lipofectamine™ 2000 transfection reagent (Invitrogen) according to the manufacturer’s instructions.

The expression vector for human *CFL2* (GV230-*CFL2*) was constructed by GeneChem (Shanghai, China), which provided GV230-empty (negative control). MGC803 cells were transfected with the GV230-*CFL2* (or GV230-empty) using jetPEI^®^DNA transfection Reagent (Polyplus, Illkirch, France) following the manufacturer’s protocol. The cells transfected with GV230-*CFL2* or GV230-empty were referred to as MGC803/GV230-*CFL2* cells or MGC803/GV230-empty cells, respectively. All cells were harvested at indicated time points after transfection and used in the subsequent experiments.

### 4.3. Quantitative Reverse Transcription Polymerase Chain Reaction (qRT-PCR)

Total RNA in cells was extracted using the TRIzol reagent (Invitrogen, Carlsbad, CA, USA) at 48 h after transfection. Reverse transcription reaction was performed using a First-Strand cDNA synthesis kit (Takara Bio, Tokyo, Japan) with 1 µg of RNA in a final volume of 20 µL. The newly synthesized cDNA was amplified by quantitative PCR, and the analysis was carried out using SYBR Premix Ex TaqII (Takara Bio, Tokyo, Japan). Reactions were processed and analyzed on an ABI 7500 qRT-PCR system (Applied Biosystems, Carlsbad City, CA, USA). Data were analyzed according to the comparative *C*_t_ (2^−∆∆*C*t^) method and normalized to human *GAPDH* or *U6* expression. Primer information is listed in Table 2.

### 4.4. Western Blotting Analysis

At 48 h after transfection, whole-cell protein extraction was performed by lysing cells in radioimmunoprecipitation assay (RIPA) lysis buffer (Santa Cruz, CA, USA). The proteins were quantified by using a BCA reagent kit (Beyotime, Shanghai, China). Proteins were separated by sodium-dodecyl sulfate polyacrylamide gel (12%) electrophoresis, and then transferred onto polyvinylidene fluoride (PVDF) membranes (Millipore, Bedford, MA, USA). The membranes were blocked with 5% non-fat milk in TBST buffer. The membranes were then immunoblotted with primary antibodies: rabbit anti-*S100A4* antibody (1:500 dilution; Abcam, Shanghai, China); rabbit anti-Cofilin 2 antibody (1:100 dilution; Abcam, Shanghai, China), and mouse anti-β-actin antibody (1:2000 dilution; Protein-tech). After washing, the membranes were exposed to peroxidase-conjugated secondary antibody (goat anti-rabbit IgG or goat anti-mouse IgG) (1:2000 dilution; Beijing Zhongshan Golden Bridge Biotechnology Co., Ltd., Beijing, China). Immunoreactive bands were detected using the chemiluminescence solvent (Thermo Scientific, Waltham, MA, USA) and visualized with Micro Chemi (DNR Bio-Imaging Systems, Jerusalem, Israel). The experiments were repeated three times.

### 4.5. Dual Luciferase Reporter Assay

The binding site of miR-3189-3p on the 3′-UTR of the *CFL2* mRNA was predicted by miRanda (http://www.microrna.org/) and TargetScan (http://www.targetscan.org). The fragment containing the wild-type or mutated binding site was cloned downstream of the firefly luciferase coding sequence in the GV272 vector by GeneChem (Shanghai, China), and were named as wt-*CFL2*-3′-UTR-reporter and mut-*CFL2*-3′-UTR-reporter, respectively. For the luciferase assay, HEK293T or MGC803 cells were grown in 24-well plates to 70% confluence, and the miR-3189-3p mimics or miR-3189-3p NC were co-transfected with reporter vectors into HEK293T or MGC803 cells using Lipofectamine™ 2000 (Invitrogen, Carlsbad, CA, USA). The luciferase activities were detected using a Dual luciferase kit (Promega, Madison, WI, USA) at 48 h after transfection, and Renilla luciferase was used as normalization control. The assays were performed independently in triplicate.

### 4.6. Cell Proliferation

At 6 h after transfection, the cells were seeded at a density of 1500 cells per well in 96-well plates and incubated at 37 °C for 24, 48, 72, or 96 h. Cell proliferation was measured using the Cell Counting Kit-8 (CCK8) (Dojindo, Rockville, MD, USA) according to the manufacturer’s instructions. The absorbance was measured at 450 nm using a microplate reader. Experiments were carried out independently three times.

### 4.7. Transwell Assay

Cell migration was analyzed using the transwell assay (Costar, Corning Inc., Corning, NY, USA). At 48 h after transfection, cells were resuspended in serum-free DMEM and plated in the upper chamber of 8-µm pore transwell apparatus at a density of 3 × 10^4^ cells per well. The lower chamber was filled with 650 μL of DMEM supplemented with 10% fetal bovine serum. After incubation for 24 h, non-migrated cells were removed from the upper surface of the insert membrane using a cotton swab, whereas the migrated cells on the lower surface were fixed with 4% paraformaldehyde, stained with hematoxylin and eosin, and then photographed. Five random fields were analyzed for each chamber to count the number of the cells under microscope. The experiments were repeated three times.

### 4.8. Wound Healing Assay

At 48 h after transfection of related oligonucleotides or vectors, when MGC803 cells reached approximately 100% confluence in 6-well plates, a wound was created using a 200 μL pathogen-free pipette tip in the confluent monolayer in each well. After being washed with phosphate-buffered saline, the cells were then cultured with serum-free DMEM medium for an additional 24 h. The wound widths were photographed and measured under a microscope in five random fields at 0 h and 24 h after wounding. The cell migration rate = (width at 0 h − width at 24 h)/width at 0 h.

### 4.9. Kaplan–Meier Plotter

The Kaplan–Meier plotter (http://kmplot.com/analysis/) is a comprehensive online platform that includes data from 1,065 patients with GC with a mean follow-up of 33 months [39]. The samples were obtained from six independent datasets (GSE14210, GSE15459, GSE22377, GSE29272, GSE51105, and GSE62254) which were downloaded from the Gene Expression Omnibus (Affymetrix microarrays), among them GSE62254 had markedly different characteristics than the other datasets and was excluded from the cross-validation analysis. To assess the prognostic value of *CFL2*, the patient samples (*n* = 593) were divided into two cohorts according to the median expression of the gene (high vs. low expression). The OS and FP of patients with GC were analyzed using a Kaplan–Meier plot, the HR with 95% CI, and the log rank *p* value were calculated and displayed.

### 4.10. Statistical Analysis

Statistical analysis was carried out using Student’s *t*-test and one-way analysis of variance (ANOVA) using Graphpad 6.0 software and the Statistical Package for the Social Sciences (SPSS Inc., Chicago, IL, USA), where *p* < 0.05 was considered to indicate statistical significance.

## Figures and Tables

**Table 1:** The nucleotides used for cell transfection.

Name of Short Nucleotides	Sequences
*S100A4*-siRNA	5′-GCAUCGCCAUGAUGUGUAATT-3′
	5′-UUACACAUCAUGGCGAUGCTT-3′
*CFL2*-siRNA	5′-GCAAGUAAAUGGCUUGGAUTT-3′
	5′-AUCCAAGCCAUUUACUUGCTT-3′
hsa-miR-3189-3p mimics	5′-CCCUUGGGUCUGAUGGGGUAG-3′
	5′-ACCCCAUCAGACCCAAGGGUU-3′
Negative Control (NC)	5′-UUCUCCGAACGUGUCACGUTT-3′
	5′-ACGUGACACGUUCGGAGAATT-3′
hsa-miR-3189-3p inhibitor	5′-CUACCCCAUCAGACCCAAGGG-3′
hsa-miR-3189-3p inhibitor NC	5’-CAGUACUUUUGUGUAGUACAA-3’

*S100A4*-siRNA, *CFL2*-siRNA, and hsa-miR-3189-3p mimics use the same NC.

**Table 2:** The primers used for qRT-PCR analysis.

Gene	Primer Sequence (5′–3′)
*S100A4*	F: CCCTGGATGTGATGGTGT
	R: GTTGTCCCTGTTGCTGTC
*GDF15*	F: CTCCAGATTCCGAGAGTTGC
	R: AGAGATACGCAGGTGCAGGT
*pri-miR-3189*	F: CAAGCAGCCCCCATATCTAA
	R: CCAAGGGGATCCAGGATATT
*miR-3189-3p*	F: ATGCTGCCCTTGGGTCTG
	R: CACTTCCTCAGCACTTGTTGGTAT
*GAPDH*	F: ATCATCAGCAATGCCTCC
	R: CATCACGCCACAGTTTCC
*U6*	F: ATTGGAACGATACAGAGAAGATT
	R: GGAACGCTTCACGAATTTG

F, forward; R, reverse.

## Abbreviations

GC	gastric cancer
miRNA	microRNA
3′-UTR	3′-untranslated region
*CFL2*	cofilin 2
OS	overall survival
FP	first progression
HR	hazard ratio
95%CI	95% confidence intervals
IBMS	Institute of Basic Medical Sciences
CAMS	Chinese Academy of Medical Sciences
PUMC	Peking Union Medical College
SPSS	Statistical Package for the Social Sciences
